# Natural *Burkholderia mallei* Infection in Dromedary, Bahrain

**DOI:** 10.3201/eid1707.110222

**Published:** 2011-07

**Authors:** Ulrich Wernery, Renate Wernery, Marina Joseph, Fajer Al-Salloom, Bobby Johnson, Joerg Kinne, Shanti Jose, Sherry Jose, Britta Tappendorf, Heidie Hornstra, Holger C. Scholz

**Affiliations:** Author affiliations: Central Veterinary Research Laboratory, Dubai, United Arab Emirates (U. Wernery, R. Wernery, M. Joseph, B. Johnson, J. Kinne, Shanti Jose, Sherry Jose);; Ministry of Municipalities Affairs and Agriculture, Barbar, Bahrain (F. Al-Salloom, B. Tappendorf);; Center for Microbial Genetics and Genomics, Flagstaff, Arizona, USA (H. Hornstra);; Bundeswehr Institute of Microbiology, Munich, Germany (H.C. Scholz)

**Keywords:** bacteria, Burkholderia mallei, glanders, Bahrain, camel, dromedary, natural infection, MLVA, dispatch

## Abstract

We confirm a natural infection of dromedaries with glanders. Multilocus variable number tandem repeat analysis of a *Burkholderia mallei* strain isolated from a diseased dromedary in Bahrain revealed close genetic proximity to strain Dubai 7, which caused an outbreak of glanders in horses in the United Arab Emirates in 2004.

Glanders, a World Organisation for Animal Health (OIE)–listed disease, is a contagious, life-threatening disease of equids caused by the gram-negative bacterium *Burkholderia mallei* (*1*). Although eliminated in western Europe, glanders remains endemic to several Asian, African, and South American countries. It recently reappeared in Pakistan and Brazil in 2008 and 2009, respectively, and appeared for the first time in Kuwait and Bahrain in 2010 (*2**,**3*).

Natural *B. mallei* infections are known to occur in various mammals (e.g., cats, bears, wolves, and dogs). Camels are also susceptible to *B. mallei*, as experimental infection has demonstrated (*4**,**5*). We report a natural infection of dromedaries (*Camelus dromedarius*).

An outbreak of glanders is ongoing in equids in Bahrain (*6*). Most of the reported cases were found in Saar and Shakhoura in the Northern governorate. Samples from 4,843 horses and 120 donkeys were sent to the OIE Reference Laboratory at the Central Veterinary Research Laboratory in Dubai, United Arab Emirates. Of these samples, 45 horses with clinical signs consistent with glanders were positive by complement fixation test and were euthanized along with 4 donkeys that also had positive test results. In addition to horses and donkeys, dromedaries showed clinical signs of glanders, but *B. mallei* infection has not yet been confirmed. Here we provide evidence for a *B. mallei* infection in 1 of the diseased dromedaries.

## The Study

**Figure 1:**
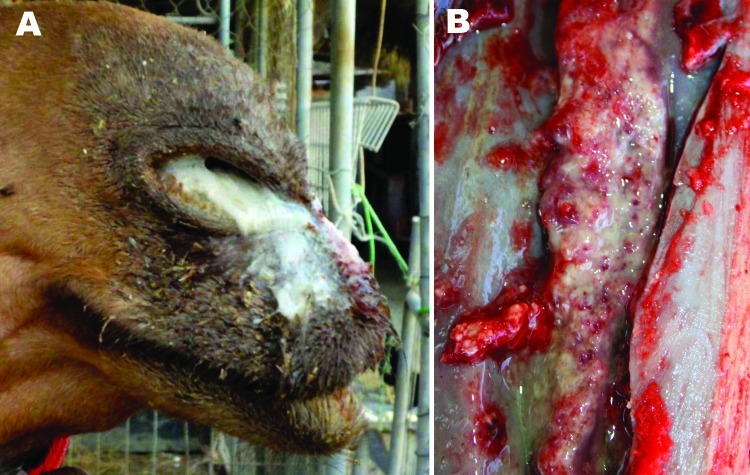
A) Severe mucopurulent discharge from both nostrils of a glanderous dromedary (*Camelus dromedarius*), Bahrain. B) Glanderous lesions in the choanae of a dromedary.

On a small private farm, 2 of 7 horses had positive serologic reactions and showed typical clinical signs of glanders. On the same premises, 6 dromedaries were kept several meters away from the sick horses in a separate enclosure. Three dromedaries that showed clinical signs of glanders, including severe mucopurulent discharge from both nostrils (Figure 1, panel A), fever, emaciation, and fatigue, died. One of these dromedaries underwent necropsy. Serum samples from this dromedary tested positive for glanders with both the OIE-acknowledged complement fixation test (titer 10+++) and with the Central Veterinary Research Laboratory–developed in-house competitive ELISA (*7*) with an inhibition of 57%.

An EDTA blood sample was incubated for 11 days in a blood culture system (Oxoid, Cambridge, UK) until it became positive. This fluid was then cultured on sheep blood agar at 37°C for 72 h. The isolate stained poorly gram-negative, was rod shaped, and tested oxidase positive. Suspected *B. mallei* colonies were analyzed with the API 20 NE-test (bioMérieux, Marcy l’Etoile, France) and were positive for nitrate, glucose assimilation, arginine dehydrolase (after 4 days of incubation), N–acetyl glucosamine, and potassium gluconate. The API 20 NE-test identified the colonies as *B. mallei* because the same API ID number (1140504) occurred as in the previously isolated Dubai 7 strain (*1*).

During necropsy, typical glanderous lesions in the lung, choanae, and nasal septae were observed. Golf ball–sized reddish-gray nodules resembling tubercles with a central gray necrotic zone were detected in the lungs. In the choanae and nasal septae, stellate scars, ulcers, and honeycomb necrotic patches covered with yellow pus (Figure 1, panel B) were seen. Glanderous lesions were absent from other organs.

The presence of *B. mallei* in lung and choanae specimens was examined by using standard culturing techniques as described by Wittig et al. (*1*). For bacterial growth, sheep blood agar plates were incubated at 37°C for 72 h. *B. mallei* was directly isolated from the pus, which had accumulated in the choanae, but not from nasal and eye swabs and not from the lung lesions. However, the tissue samples were stored at –20°C for >20 days before incubation.

For molecular analysis, cultivated bacteria were resuspended in saline and inactivated at 98°C for 20 min. Total DNA was extracted by using the DNA-purification Kit (QIAGEN, Hilden, Germany). Sequence analysis of the 16S rRNA (1,400 pb) gene displayed the *B. mallei*–specific single nucleotide polymorphism that differentiates *B. mallei* from *B. pseudomallei* (*8*) (not shown). *B. mallei* was further confirmed by multilocus sequence typing displaying the *B. mallei*–specific sequence type 40 (alleles 1, 12, 3, 4, 1, 18, 1), as previously described by Godoy et al. (*9*).

**Figure 2:**
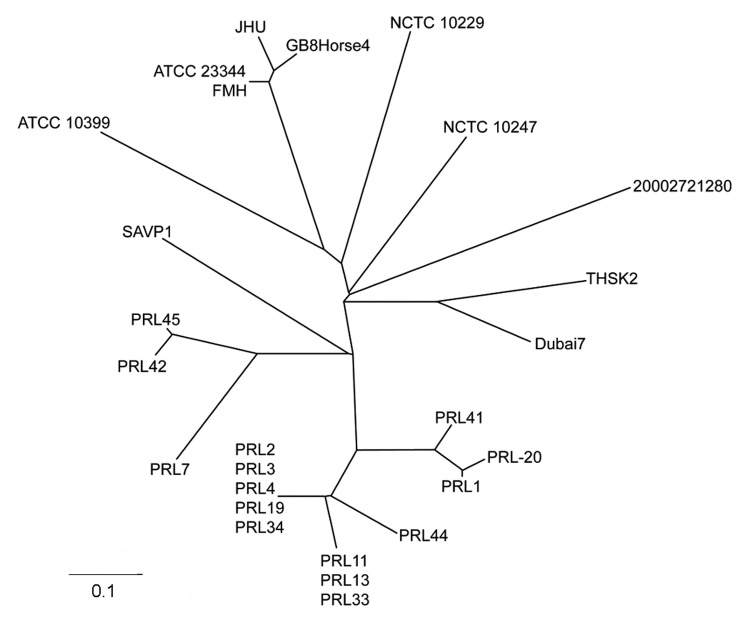
Unrooted neighbor-joining tree based on 23 variable number tandem repeat loci demonstrating the genetic relationship of the camel strain (THSK2) to other existing strains of *Burkholderia mallei*. The most closely related *B. mallei* strain to THSK2 is Dubai 7, which was isolated from a horse in the United Arab Emirates in 2004. Scale bar represents 0.1 changes.

Multilocus variable number tandem repeat analysis based on 23 different loci (*10*) was used for further subtyping through sequencing of the variable number tandem repeat regions (Table A1). Phylogenetic analysis of these data was performed as described by Hornstra et al. (*10*) and compared with existing *B. mallei* strains (Figure 2). In this analysis, the strain (THSK2) isolated from the dromedary clustered with *B. mallei* strain Dubai 7 (Figure 2) that had been isolated from a horse in the United Arab Emirates (*11*).

## Conclusions

Old World camels, the dromedary (*C. dromedarius*), and the Bactrian camel (*C. bactrianus*) are susceptible to *B. mallei* (glanders) and *B. pseudomallei* (melioidosis) infection (*12**,**13*). However, reports of *B. mallei* infection in dromedaries have described artificial infections (*4**,**5*). We report natural *B. mallei* infection in a dromedary that occurred during a glanders outbreak in horses.

Clinical signs as well as gross pathologic and microscopic lesions of the diseased dromedary were similar to changes seen in equids. These changes were dominated by severe mucopurulent nasal discharge, nodules and ulcers with pus in the choanae and nasal septae, and granulomas in the lungs that resembled tubercle lesions (pseudo tubercles).

*B. mallei* was isolated from venous blood, indicating septicemia. The pathogen was also directly isolated from the pus, which had accumulated in the choanae, but not from nasal and eye swabs and, unexpectedly not from the lung lesions. A possible explanation for the failure to isolate *B. mallei* from the nasal swabs was the heavy growth of various other contaminating bacteria because no selective culture medium exists for *B. mallei*. It could also be explained by storage of the samples at –20°C for >20 days, which probably destroyed the bacteria.

The genetic relatedness of the strain isolated from the dromedary to the strain isolated in 2004 from horses in the United Arab Emirates suggests that this strain might be endemic to this region. It also appears to be genetically distinct from a recent outbreak in Pakistan, demonstrating the persistence of multiple strains on a larger geographic scale. Isolation of this pathogen from both camels and horses poses new challenges to the international trade of equids from and to countries where camels are raised.

## Appendix

**Table A1:** Comparison of 23-locus VTNR data for 2 new isolates of *Burkholderia mallei*, THSK2 and Dubai 7, with data for previously published strains*

Isolate	Type of animal	Location	Collection year	VNTR loci +																						
				20	389	397	857	933	1500	1690	1764	1788	1934	2050	2065	2170	2341	2356	2445	2666	2815	3091	3145	315k	3564	3652
PRL1	Donkey	Pakistan	2002	9	7	13	10	6	NA	10	8	5	NA	17	12	18	3	6	3	2	6	10	5	23	23	6
PRL2	Horse (mare)	Pakistan	1999	9	9	13	10	10	NA	10	8	5	NA	20	13	24	3	6	3	2	6	10	5	24	23	13
PRL3	Horse (gelding)	Pakistan	2005	9	9	13	10	10	NA	10	8	5	NA	20	13	24	3	6	3	2	6	10	5	24	23	13
PRL4	Horse (gelding)	Pakistan	2005	9	9	13	10	10	NA	10	8	5	NA	20	13	24	3	6	3	2	6	10	5	24	23	13
PRL7	Horse	Pakistan	2000	10	6	12	11	8	NA	10	8	6	NA	26	10	25	3	9	3	2	6	9	8	19	23	12
PRL11	Horse	Pakistan	1999	9	9	13	10	10	NA	10	8	5	NA	19	13	21	3	6	3	2	6	10	5	24	23	12
PRL13	Horse	Pakistan	1999	9	9	13	10	10	NA	10	8	5	NA	19	13	21	3	6	3	2	6	10	5	24	23	12
PRL19	Horse (gelding)	Pakistan	2005	9	9	13	10	10	NA	10	8	5	NA	NA	13	24	3	NA	3	2	6	10	5	24	NA	13
PRL20	Horse (gelding)	Pakistan	2005	9	7	13	10	6	NA	10	NA	5	NA	17	12	18	3	6	3	2	6	10	5	23	23	6
PRL33	Donkey	Pakistan	2007	9	9	13	10	10	NA	10	8	5	NA	19	13	21	3	6	3	2	6	10	5	24	23	12
PRL34	Donkey	Pakistan	2007	9	9	13	10	10	NA	10	8	5	NA	20	13	24	3	6	3	2	6	10	5	24	23	13
PRL41	Mule	Pakistan	2006	9	7	13	10	6	NA	10	8	5	NA	20	12	19	3	6	3	2	6	10	5	23	23	6
PRL42	Mule	Pakistan	2007	10	6	12	11	8	NA	10	8	5	NA	18	9	26	3	8	3	2	6	9	8	14	23	11
PRL44	Mule	Pakistan	2007	9	9	13	9	10	NA	10	8	5	NA	22	13	21	3	6	3	2	6	10	5	23	23	13
PRL45	Mule	Pakistan	2007	10	6	13	11	8	NA	10	8	5	NA	18	9	26	3	8	3	2	6	9	8	14	23	11
200272 1280	UNK	UNK	UNK	11	7	13	10	14	4	7	15	5	19	17	7	22	3	7	NA	2	4	NA	9	19	16	15
ATCC 23344	NA (human)	China/ Burma	1944	30	6	9	13	9	4	9	8	5	20	20	13	NA	3	9	3	2	4	9	7	24	21	4
FMH	NA (human)	China/ Burma	UNK	30	6	9	13	9	4	9	8	5	20	20	13	NA	3	9	3	2	4	9	7	24	21	4
JHU	NA (human)	China/ Burma	UNK	30	6	9	13	9	4	9	NA	5	20	20	12	NA	3	9	3	2	4	9	7	24	21	4
GB8 Horse 4	Horse	China/ Burma	UNK	34	6	9	13	9	4	9	NA	5	20	20	13	NA	3	9	3	2	4	9	7	24	21	4
NCTC 10247	UNK	Turkey	1960	10	7	17	10	6	4	9	NA	5	17	16	13	23	3	8	3	2	4	10	9	23	23	12
NCTC 10229	UNK	Hungary	1961	12	12	17	10	11	4	9	16	6	18	20	9	14	3	8	3	2	4	9	10	29	23	14
SAVP1	UNK	India	UNK	10	NA	NA	NA	5	NA	9	8	5	NA	21	11	23	3	7	3	2	5	9	5	15	23	9
ATCC 10399	Horse	China	1942	19	5	9	11	11	4	9	NA	5	15	27	6	32	3	12	3	2	3	9	8	8	24	8
THSK2	Dromedary	Bahrain	2010	18	5	13	12	3	4	9	8	5	NA	16	9	25	NA	7	3	2	4	10	10	18	21	14
Dubai 7	Horse	Dubai	2004	19	5	13	12	3	4	9	8	5	NA	19	10	29	3	7	3	2	4	10	10	10	23	10

*For the 23 loci, data are given as repeat copy number. NA indicates no amplification (failures), likely because of mutations in the priming sites or incomplete sequencing of VNTR regions and does not necessarily indicate absence of a VNTR region. VNTR data for all strains except THSK2 and Dubai 7 from Hornstra et al. 2009 (*10*). VNTR, variable-number tandem repeat; UNK, unknown.
